# Seroepidemiology of toxoplasmosis in pregnant women and detection of infection acquired during pregnancy in Cotonou, Benin[Fn FN1]

**DOI:** 10.1051/parasite/2023040

**Published:** 2023-10-19

**Authors:** Richard Amagbégnon, Celia Dechavanne, Magalie Dambrun, Urielle Yehouénou, Noé Akondé, Florence Migot-Nabias, Aretas Babatoundé Nounnagnon Tonouhéwa, Azra Hamidović, Nadine Fievet, Angéline Tonato-Bagnan, Aurore Ogouyemi-Hounto, Maroufou Jules Alao, Marie-Laure Dardé, Aurélien Mercier, Dorothée Kindé-Gazard

**Affiliations:** 1 Centre Hospitalier Universitaire de la Mère et de l’Enfant-Lagune (CHU-MEL) 01 BP 107 Cotonou Bénin; 2 Institut de Recherche Clinique du Bénin (IRCB) Abomey-Calavi Benin; 3 Université Paris Cité, MERIT, IRD Paris France; 4 Unité de Recherche sur les Maladies Transmissibles (URMAT), Université d’Abomey-Calavi 01 BP 2009 Cotonou Bénin; 5 Inserm U1094, IRD U270, Univ. Limoges, CHU Limoges, EpiMaCT - Epidémiologie des maladies chroniques en zone tropicale, Institut d’Epidémiologie et de Neurologie Tropicale, OmegaHealth Limoges France; 6 Université d’Abomey-Calavi (UAC), Faculté des Sciences de la Santé (FSS) Bénin; 7 Service de Microbiologie du Centre National Hospitalier Universitaire – Hubert Koutoukou MAGA (CNHU-HKM) de Cotonou Bénin; 8 Centre National de Référence (CNR) sur la toxoplasmose/Toxoplasma Biological Resource Center (BRC), CHU de Limoges 87042 Limoges France

**Keywords:** *Toxoplasma gondii*, Seroprevalence, Survey, Pregnancy, Maternal primary infection, Benin

## Abstract

Assessing the prevalence of toxoplasmosis in pregnant women and the associated risk factors is the first step in defining policy for the prevention of congenital toxoplasmosis in a given population. An epidemiological study was conducted during prenatal consultations at the CHU-MEL of Cotonou (Benin) between September 2018 and April 2021 and recruited 549 pregnant women to determine the seroprevalence and potential factors associated with *Toxoplasma gondii* infection. *Toxoplasma gondii* IgG/IgM antibodies were detected using an enzyme-linked fluorescence assay (ELFA) technique, an IgG avidity test and an IgG/IgM comparative Western blot to diagnose the maternal toxoplasmosis serological status, the possibility of an infection acquired during pregnancy and congenital infection, respectively. Concomitantly, the participants answered a questionnaire investigating potential risk factors. Toxoplasmosis seroprevalence was estimated at 44.4% (95% CI 40.3–48.6) and the factors significantly associated with *T. gondii* seropositivity were: age over 30 years, multigravid women and contact with cats. The possibility of an infection acquired during the periconceptional period or the first trimester of pregnancy concerned six women [1.1% (95% CI 0.5–2.0)]. However, due to the low rate of serological controls in seronegative women, a significant proportion of women first tested during the 3rd trimester of pregnancy, and an insufficient sample size, the incidence of primary infection during pregnancy could not be determined. No cases of congenital transmission occurred in the newborns from the suspected cases of primary infection.

## Abstract (fr)

L’évaluation de la prévalence de la toxoplasmose chez la femme enceinte et des facteurs de risque associés est la première étape pour définir une politique de prévention de la toxoplasmose congénitale dans une population donnée. Une étude épidémiologique a été menée lors des consultations prénatales au CHU-MEL de Cotonou (Bénin) entre septembre 2018 et avril 2021 et a recruté 549 femmes enceintes pour déterminer la séroprévalence et les facteurs potentiels associés à l’infection à *Toxoplasma gondii.* Les anticorps IgG / IgM de *T. gondii* ont été détectés à l’aide d’une technique ELFA, du test d’avidité IgG et du Western blot comparatif IgG / IgM pour diagnostiquer respectivement le statut sérologique de la toxoplasmose maternelle, la possibilité d’une infection acquise pendant la grossesse et l’infection congénitale. Parallèlement, les participants ont répondu à un questionnaire portant sur les facteurs de risque potentiels. La séroprévalence de la toxoplasmose a été estimée à 44,4 % (IC 95 % 40,3–48,6) et les facteurs significativement associés à la séropositivité pour *T. gondii* étaient l’âge supérieur à 30 ans, la multigravidité et les contacts avec les chats. La possibilité d’une infection acquise pendant la période périconceptionnelle ou le premier trimestre de la grossesse concernait six femmes [1,1 % (IC 95 % 0,5–2,0)]. Cependant, en raison du faible taux de contrôles sérologiques chez les femmes séronégatives, d’une proportion importante de femmes testées pour la première fois au cours du 3ème trimestre de la grossesse et d’une taille d’échantillon insuffisante, l’incidence de la primo-infection pendant la grossesse n’a pas pu être déterminée. Aucun des enfants nés des six femmes suspectes de primo-infection en cours de grossesse n’a présenté d’infection congénitale.

## Introduction

*Toxoplasma gondii* is the causative agent of a widespread parasitic zoonosis with more than a third of humans infected worldwide [12, 20, 27, 29]. This parasitosis is a neglected health problem for populations in developing countries living in hot and humid climates, where public health infrastructure is not optimal [12]. The millions of highly resistant oocysts excreted in the environment by the definitive host (i.e., domestic cat in urban areas) and the cysts contained in undercooked meats are the main sources of contamination [11, 15]. Usually asymptomatic, *T. gondii* infection can cause retinochoroiditis, or more rarely severe systemic infection in immunocompetent patients, especially when infected with virulent strains [19, 27], while immunocompromised patients can develop cerebral and extra-cerebral toxoplasmosis [36]. Congenital infection can cause spontaneous abortion, severe neurological damage, in particular hydrocephalus, intracranial calcifications and microcephaly, and ocular damage, with retinochoroiditis detectable at birth or appearing late or several years after birth [4, 5, 27, 30]. In particular, those acquired in the first and second trimesters are likely to affect the vital prognosis of the infected foetus or newborn.

The proportion of women at risk of infection and risk factors in a given population are the true indicators of interest when making decisions concerning the design of a congenital toxoplasmosis prevention programme and targeting relevant actions. In different regions of the world, various factors play a role in the transmission of toxoplasmosis. In Africa, in addition to the consumption of undercooked infected meat, older age and agricultural activity are the main factors associated with toxoplasmosis [34]. In some areas of West Africa, education, urban residence, and consumption of either pork, beef, mutton, wild meat, or poultry have been identified as potential factors associated with *T. gondii* infection in pregnant women [3]. Previous studies in urban Benin reported older age and urban residence as risk factors, but in rural areas, consumption of raw vegetables was associated with *T. gondii* seropositivity [22, 31]. Worldwide meta-analytic studies over nearly four decades have estimated an overall incidence of primary *Toxoplasma* infection in pregnant women at 1.1% [30]. This incidence increased to 1.6% in African regions [30]. Several factors influence the occurrence and severity of congenital toxoplasmosis (CT), including gestational age at primary infection, strain virulence in relation to parasite genotype, parasite load during the period acute infection, delay in treatment initiation after acute maternal infection and lack of educational approaches [27, 30, 38]. This multiplicity of determinants shows that the clinical and epidemiological profile of the disease is not uniform and that the public health impact of CT needs to be assessed country by country, or even by region in some countries [24].

The global extent of CT was assessed in 2013 from data available for every country in the world [35]. In Benin, its prevalence was estimated at 0.34% or 340 infected newborns per 100,000 births, and was considered the highest out of 45 African countries [35]. A meta-analytical study of toxoplasmosis in pregnant women performed on data from studies conducted in Benin over the past three decades estimated an overall seroprevalence of 47% (95% CI 42–54; *p* < 0.05) [33]. In a setting where surveillance of seronegative pregnant women is not systematic, there are few objective data to estimate the proportion of pregnant women at risk for toxoplasmosis. The main objective of this study was to determine the seroprevalence of *T. gondii* in an urban area in Benin, West Africa, in a population of pregnant women recruited during prenatal consultation at the Centre Hospitalier Universitaire de la Mère et de l’Enfant-Lagune. The specific objective was to indicate potential factors associated with toxoplasmosis in pregnant women. This epidemiological survey offers the opportunity to detect suspected cases of primary infection acquired during pregnancy.

## Materials and methods

### Study location

The study took place at the Centre Hospitalier Universitaire de la Mère et de l’Enfant-Lagune (CHU-MEL) in Cotonou, Benin. This hospital is the reference centre in terms of health care for women of childbearing age and children. It treats women from different backgrounds from Cotonou, the economic capital of Benin, and the neighbouring departments of Atlantique and Ouémé. This cosmopolitan and multi-ethnic city covers an area of 79 km^2^ and the 5th Demographic and Health Survey conducted in Benin between 2017 and 2018 reported a population of 15,928 women aged 15–49 years [17, 18].

### Type, period and study population

This prospective and analytical study was conducted from September 2018 to April 2021. It was based on an epidemiological investigation of toxoplasmosis in pregnant women and surveillance of primary infection during pregnancy, leading to neonatal and postnatal CT workups.

The sample size was determined using the formula proposed by Giezendanner in 2012 [14]:

$$n=\frac{t^{2}N}{t^{2}+{\left(2e\right)}^{2}(N-1)},$$

*N*: size of the parent population (or parent population, or reference population, or original population).
*n*: sample size for a very large parent population.
*t*: margin coefficient deduced from the confidence rate “*s*”.
*e*: margin of error given for the quantity we want to estimate.
*p*: proportion of the elements of the parent population having a given property (when *p* is unknown, we used *p* = 0.5).

The size of the maternal population was estimated based on statistics provided by the Division of Patient Management and Statistics at CHU-MEL during the period 2013–2016. During these years, the average annual number of prenatal consultations performed was 8110. Considering that a woman should have an average of 4 consultations during pregnancy, this represents approximately 2028 pregnant women consulting each year. The duration of recruitment of participants for the study was estimated at two years, so the total population N is estimated at 4056 pregnant women.

For the sample size (*n*) with a risk of error (*e*) of 5%, a confidence level of 99%, the deduced margin coefficient (*t*) is 2.57. The size “*n*” calculated according to the above formula is estimated to be 571 pregnant women.

### Inclusion and exclusion criteria

Pregnant women who met the inclusion criteria (and were free of severe medical conditions such as diabetes, hypertension or gestational malaria) were informed of the study during a routine prenatal consultation. They were enrolled after they provided voluntary written consent to a blood sample to assess their serological status for *Toxoplasma* after filling in a questionnaire. Those who were in their first or second trimester were informed that they would be invited to be retested 3 months later, free of charge, if found to be seronegative. Participating pregnant women could subsequently inform the team if they wished to withdraw for the rest of the study. Nevertheless, their data would be used to assess risk factors.

### Survey questionnaire

The participants were invited to answer the survey questionnaire (Supplementary material) during direct exchange with the interviewer. Demographic data included age, gestational age and place of residence. Socioeconomic data provided information on the educational level and employment status of the participant and her spouse and their marital status. The level of understanding of toxoplasmosis was evaluated in light of patient awareness of different disease facets, (mode of contamination, types of complications and preventive measures) as well as the sources of information. Food consumption, eating habits and lifestyle were recorded. Contact with cats was identified by the presence of cats in the participant’s home or workplace. The participant’s HIV immunological profile provided information on the immune status.

### Biological material, collection and serological analysis

Venous blood (5 mL) was collected in a dry tube from each study participant after consent and questionnaire were completed. These samples were sent to the medical biology laboratory and centrifuged at 3000 rpm for 5 min. Sera were aliquoted and stored at −20 °C before serology was performed.

Serological diagnosis of toxoplasmosis was performed using an enzyme-linked fluorescence assay (ELFA): VIDAS Toxo IgG II, VIDAS Toxo IgM on a mini Vidas automat (BioMérieux, Marcy l’Étoile, France).

- For IgG, a titre less than 4 IU/mL was considered negative, between 4 and 7 IU/mL uncertain, and equal to or greater than 8 IU/mL positive;
- For IgM, an index lower than 0.55 was considered negative, between 0.55 and less than 0.65 uncertain, and equal to or greater than 0.65 positive.

For cases of suspected primary infection during pregnancy (presence of IgG and IgM), an IgG avidity test was performed to help date the infection (VIDAS Toxo Avidity, BioMérieux). An avidity index strictly less than 0.2 indicates low avidity IgG, between 0.2 and less than 0.3 intermediate avidity IgG, and equal to or greater than 0.3 high avidity IgG. High avidity indicates an infection lasting more than four months and low or intermediate avidity indicates that an infection less than four months cannot be excluded. IgG avidity according to gestational period excludes an infection contracted before pregnancy and identifies an infection possibly acquired during pregnancy. Pregnant women with suspected primary infection were managed until delivery with spiramycin chemoprophylaxis, ultrasound monitoring per trimester and neonatal CT diagnosis. Participants were aware that they would not be charged for any of the tests and treatments.

### Neonatal and postnatal work up for congenital toxoplasmosis

Neonatal and postnatal work up for CT was performed in all children born to women suspected of infection acquired during pregnancy.

Comparative IgG and IgM Western blot (WB) (LDBIO *Diagnostics*, Lyon, France) were performed on maternal and neonatal blood samples between Day 3 and Day 10 post-delivery. A positive comparative WB result was indicative of congenital infection. A negative WB result did not automatically rule out congenital infection.

Toxoplasmosis serology was performed at birth (Day 3–Day 10) by the same method (ELFA VIDAS Toxo IgGII, VIDAS Toxo IgM) with the same thresholds as used for adults. Each child was tested for specific IgG and IgM antibody kinetics every three months for the first nine months after birth. A significant increase in IgG antibodies suggested congenital infection. Progressive disappearance of IgG antibodies indicated the absence of congenital infection.

### Statistical tests and analyses

Data were coded (including anonymisation) and recorded on the survey questionnaire designed by Epi info V 7.2.4.0 software and then imported into STATA 12 software (Stata Corp, College Station, TX, USA) for statistical analysis. Descriptive analyses of sociodemographic characteristics and serological profiles were performed with a risk of 0.05 and a 95% confidence interval. For statistical analysis of risk factors, uncertain IgG serological results were excluded. A logistic regression model was used to examine the association between *T. gondii* seropositivity and these factors. Univariate regression was used to examine the association between IgG seropositivity at inclusion of the participants and the different explanatory variables. The multivariate regression model was constructed from the univariate study variables with probability *p* less than or equal to 0.2 to highlight potential factors associated with the occurrence of anti-*Toxoplasma* antibodies. Crude odds ratios (ORs) from univariate models and adjusted odds ratios (mORs) from multivariate models were calculated.

### Ethical considerations

The study received authorisation from CHU-MEL and approval from scientific experts and the ethics and research committee of the Institute of Applied Biomedical Sciences of the Faculty of Health Sciences of the University of Abomey-Calavi (UAC) under No. 120 of 20 August 2018. This authorisation was renewed once to cover the entire study period. All participants gave their free and informed written consent. The data for each participant were kept strictly anonymous.

## Results

### Characteristics of the study population

**Figure 1:**
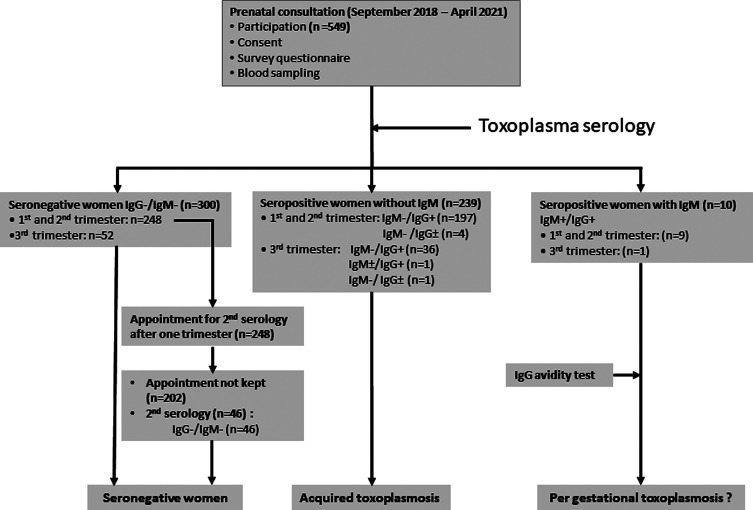
Flow chart of CHU-MEL toxoplasmosis study. IgM: immunoglobin M, IgG: immunoglobin G, +: Positive, −: Negative, ±: Uncertain.

During the study, we were able to enrol 549 pregnant women admitted to the CHU-MEL for routine prenatal consultations, which represented our study sample size (Table 1 and Fig. 1). The concomitant occurrence of the study with the COVID-19 pandemic period in 2020 partially explains why we did not reach the calculated sample size of 571 women. In addition, we received a significant number of pregnant women (*n* = 44) who were excluded from the study after inclusion because they had high blood pressure, diabetes, or gestational malaria or decided to withdraw from the study.

**Table 1:** Population characteristics (*n* = 549).

Variables	Number of women (*n*)	Percentage % (CI 95%)
Age (years)		
15–24	150	27.3 (23.8–31.2)
25–29	174	31.7 (27.9–35.7)
30–47	225	41.0 (36.8–45.0)
Education		
University education	229	41.7 (37.6–45.9)
Secondary education	185	33.7 (29.8–37.7)
Primary education	69	12.6 (10.0–15.6)
Illiterate	66	12.0 (9.5–15.0)
Occupation		
High-level	119	21.7 (18.4–25.3)
Office workers	124	22.6 (19.3–26.2)
Shopkeeper/salesperson	73	13.3 (10.7–16.4)
Crafts woman	153	27.8 (24.3–31.7)
Housewife	80	14.6 (11.8–17.7)
Cohabitation with partner		
Yes	479	87.2 (84.2–89.8)
No	70	12.8 (10.2–15.8)
Department of residency		
Atlantique	198	36.1 (32.1–40.2)
Littoral	296	53.9 (49.7–58.0)
Ouémé	55	10.0 (7.8–12.8)
Trimester at screening		
1st trimester	152	27.7 (24.1–31.6)
2nd trimester	306	55.7 (51.5–59.8)
3rd trimester	91	16.6 (13.7–19.9)
Gravidity		
Primigravidae	174	31.7 (27.9–35.7)
Multigravidae	375	68.3 (64.3–72.1)
HIV status		
Negative	527	96.0 (94.0–97.3)
Positive	12	2.2 (1.2–3.8)
Not known	10	1.8 (1.0–3.3)
Awareness of toxoplasmosis		
No	491	89.4 (86.6–91.7)
Yes	58	10.6 (8.3–13.4)

The mean age of this population was 28.3 ± 5.7 years, 90% of whom resided in the Littoral and Atlantique departments. Considering gestational age, 27.7% (95% CI 24.1-31.6), 55.7% (95% CI 51.5–59.8) and 16.6% (95% CI 13.7–19.9) were included in the study during the 1st, 2nd and 3rd trimester of their pregnancy, respectively. Most participants [89.4% (95% CI 86.6–91.7)] had no knowledge of the infection, including modes of transmission, clinical complications and prevention strategies of toxoplasmosis in pregnant women and newborns. A majority of the pregnant women [96% (95% CI 94.0–97.3)] were HIV negative.

### Serological status at the first test

A total of 549 sera samples were tested for *T. gondii* specific IgG and IgM antibodies. Sera samples from 300 participants were negative for both antibodies, representing 54.6% (95% CI 50.5–58.8) of non-immune pregnant women and the presence of IgG antibodies in 244, indicated a seroprevalence of 44.4% (95% CI 40.3–48.6). For the seropositive samples, 10 [1.8% (95% CI 1.0–3.3)] and 5 [1.0% (95% CI 0.4–2.1)] were seropositive for both IgG/IgM and uncertain for IgG antibodies, respectively. None of the participants had a serological profile with positive IgM and negative IgG antibodies (Fig. 1).

### Surveillance of infections acquired during the gestational period

At participant inclusion, 300 pregnant women were seronegative (IgM−/IgG−), five had equivocal IgG, and one had equivocal IgM. Of these pregnant women, 253 were included during the first and second trimesters and 53 were included during the third trimester of pregnancy. Those included in the first and second trimester of pregnancy (*n* = 253) were invited after one trimester for a second serology. Fifty of them (19.8%) returned for this second serology. No participants with uncertain IgG antibodies returned for the control (Fig. 1). No seroconversion was detected.

Both specific IgG and IgM antibodies were detected in 10 pregnant women (Table 2). In six of them, the avidity index could not rule out that infection occurred early during pregnancy, because i) the index was low in two participants (cases No. 1 and 2) and intermediary in one (case No. 3), and ii) a high index was found in three patients sampled after 20 weeks of gestation (cases No. 4, 5, and 6). Ultrasound monitoring revealed some non-specific suggestive signs in 50% of cases (cases No. 1, 2, and 5). Placental calcification was reported in three cases, one of which had a completely calcified placenta at 37 days gestation.

**Table 2:** Description of the profile of the 10 pregnant women with positive IgM and IgG.

Suspected cases of primary infections	Gestational age (WA)	Pregnancy trimester	IgG avidity	Interpretation	Primary infection	Ultrasound monitoring
Case No. 1	17	2	Low	Possible infection during the 1st trimester	Selected	37WA: CPC
Case No. 2	19	2	Low	Possible infection during the 1st trimester	Selected	34WA: PPC
Case No. 3	21	2	Intermediate	Possible infection during the 1st trimester	Selected	31WA: NVA
Case No. 4	31	3	High	Possible infection during the 1st trimester	Selected	35WA: NVA
Case No. 5	26	2	High	Possible periconceptional infection	Selected	32WA: PPC + PES + LA
Case No. 6	23	2	High	Possible periconceptional infection	Selected	35WA: NVA
Case No. 7	19	2	High	Pre-conceptional infection	Excluded	
Case No. 8	10	1	High	Pre-conceptional infection	Excluded	
Case No. 9	10	1	High	Pre-conceptional infection	Excluded	
Case No. 10	6	1	High	Pre-conceptional infection	Excluded	

WA: weeks of amenorrhoea, PPC: partial placenta calcification, CPC: complete placenta calcification, PES: pericardial effusion slide, LA: liver arrow, NVA: no visible anomaly.

It should be noted that for the 38 seropositive women tested for the first time in the third trimester, a per gestational infection could not be excluded due to late blood sampling (Fig. 1).

### Neonatal and postnatal work up for CT

No neosynthesis of IgG and IgM was detected at birth in the six children born to a mother for whom acute infection during pregnancy could not be ruled out. Their IgG titers decreased to undetectable levels before nine months of age, thus confirming the absence of CT for all suspected cases.

### Risk factors associated with *T. gondii* infection: univariate analysis and multivariate logistic regression

The study of risk factors was performed in 544 pregnant women. The five pregnant women with an uncertain IgG result were not included in the risk factor analysis. The results are summarised in Table 3. The univariate analysis shows that of all the risk factors studied, the age of the pregnant woman between 30 and 47 years (OR = 2.44; 95% CI 1.58–3.75; *p* < 0.01) and gravidity are highly significant and strongly associated with *T. gondii* seropositivity. This implies that pregnant women aged 30–47 years have twice the risk of having been infected with *T. gondii* compared to women aged 15–24 years. The risk is also doubled in multigravid (OR = 2.25) compared to primigravid (OR = 1) women. Contact with cats (OR = 1.57; 95% CI 1.01–2.45; *p* < 0.05) and the primary education level of pregnant women (OR = 1.75; 95% CI 1.01–3.02; *p* < 0.05) were significantly associated with *T. gondii* seropositivity with an OR > 1 risk. The other risk factors studied, i.e., the woman’s occupation, date of pregnancy, HIV seropositivity, notions of toxoplasmosis, living with a partner, department of residence, consumption of undercooked meat, poorly washed raw vegetables and undertreated water, did not appear to have a significant effect on the seroprevalence of toxoplasmosis in pregnant women in urban areas (*p* > 0.05).

**Table 3:** Univariate and multivariate analyses of risk factors (*n* = 544).

			Univariate analysis		Multivariate logistic regression	
Variables	Category	Seroprevalence IgG *n*/*N* (%)	OR (95% CI)	*p* value	mOR (95% CI)	pm value
Age (years)						
	15–24	49/148 (33.1)	1			
	25–29	72/171 (42.1)	1.47 (0.93–2.32)	0.10	1.42 (0.85–2.36)	0.18
	30–47	123/225 (54.7)	2.44 (1.58–3.75)	**<0.01** *	2.00 (1.16–3.43)	**0.01** *
Education						
	University education	91/228 (39.9)	1			
	Secondary education	87/184 (47.3)	1.35 (0.91–1.99)	0.13	1.14 (0.68–1.91)	0.62
	Primary education	36/67 (53.7)	1.75 (1.01–3.02)	**0.04** *	1.49 (0.71–3.13)	0.29
	Illiterate	30/64 (46.1)	1.29 (0.74–2.24)	0.37	1.01 (0.49–2.11)	0.97
Occupation						
	High-level	44/119 (37.0)	1			
	Office workers	60/122 (49.2)	1.65 (0.98–2.75)	0.06	1.69 (0.94–3.04)	0.08
	Shopkeeper/salesperson	31/73 (42.5)	1.26 (0.69–2.28)	0.45	1.02 (0.50–2.07)	0.96
	Crafts woman	70/150 (46.7)	1.49 (0.91–2.44)	0.11	1.17 (0.58–2.38)	0.66
	Housewife	39/80 (48.7)	1.62 (0.91–2.88)	0.10	1.45 (0.70–3.02)	0.31
Cohabitation with partner						
	Yes	211/476 (44.3)	1			
	No	33/68 (48.5)	1.18 (0.71–1.97)	0.51		
Department of residency						
	Littoral	131/293 (44.7)	1			
	Atlantique	88/198 (44.4)	0.99 (0.69–1.42)	0.95		
	Ouémé	25/53 (47.2)	1.10 (0.61–1.98)	0.74		
Trimester at screening						
	1st trimester	76/154 (49.4)	1			
	2nd trimester	128/298 (43.0)	0.83 (0.56–1.23)	0.37		
	3rd trimester	37/89 (41.6)	0.78 (0.46–1.32)	0.36		
Gravidity						
	Primigravidae	54/171 (31.6)	1			
	Multigravidae	190/373 (50.9)	2.25 (1.54–3.29)	**<0.01** *	1.67 (1.07–2.62)	**0.02** *
HIV status						
	Negative	233/522 (44.8)	1			
	Positive	9/12 (75.0)	3.72 (0.99–13.90)	**0.05** *	3.08 (0.79–12.00)	0.10
Awareness of toxoplasmosis						
	Yes	32/58 (55.2)	1			
	No	212/486 (43.6)	1.59 (0.92–2.75)	0.09	1.54 (0.85–2.78)	0.16
Contact with cats						
	No	192/448 (42.8)	1			
	Yes	52/96 (54.2)	1.57 (1.01–2.45)	**0.04** *	1.89 (1.17–3.06)	**0.01** *
Undercooked meat						
	No	110/255 (43.1)	1			
	Yes	134/289 (46.4)	1.14 (0.81–1.60)	0.45		
Poorly washed raw vegetables						
	No	163/356 (45.8)	1			
	Yes	81/188 (43.1)	0.89 (0.62–1.28)	0.55		
Poorly treated water						
	No	226/508 (44.5)	1			
	Yes	18/36 (50.0)	1.25 (0.63–2.45)	0.52		
Lack of hand washing						
	No	124/270 (45.9)	1			
	Yes	120/274 (43.8)	0.92 (0.65–1.28)	0.62		

* p-value limit or below the significance threshold 0.05.

From this univariate analysis, variables with probability *p* value less than 0.2 were retained for multivariate logistic regression following a top-down stepwise procedure (Table 3). This analysis takes into account age group, level of education of the woman, professional level, gravidity index, HIV serology, contact with cats, and awareness of toxoplasmosis. At the end of this analysis, the variables “age between 30–47 years” (mOR = 2.00; 95% CI 1.16–3.43; pm = 0.01), “multigravidae women” (mOR = 1.67; 95% CI 1.07–2.62; pm = 0.02) and “contact with cats” (mOR = 1.89; 95% CI 1.17–3.06; pm = 0.01) emerged as significantly associated with the seroprevalence of toxoplasmosis. These factors can be considered potential risk factors for *T. gondii* seropositivity in urban pregnant women.

## Discussion

Congenital toxoplasmosis is an often unrecognised public health problem in developing countries, where public health infrastructures are not optimal. Serological surveillance of infection in non-immune pregnant women is an essential step in the prevention of congenital toxoplasmosis.

The current study was conducted to assess the seroprevalence and to determine potential risk factors associated with infection among pregnant women in an urban area of Benin.

Demographic data from this study revealed that the mean age of pregnant women attending CHU-MEL was 28.3 ± 5.7 years, similar to results from previous studies in Benin with an average age of 29.5 ± 5.0 years and 28.6 ± 5.5 years, respectively for studies conducted in urban Cotonou in 1993 [28] and in a semi-rural area located 70 km from Cotonou between 2008 and 2010 [8]. In contrast, the population in the present study was relatively older than that reported in 2016 in rural Benin where the mean age was 26.6 ± 10.0 years [31]. Furthermore, in our study, nearly nine out of ten pregnant women had no understanding of toxoplasmosis. This rate is relatively high in Benin, as in many neighbouring countries: 99.5% in rural areas in Benin, 86.0% in Togo and 86.9% in Nigeria [10, 23, 31]. These figures reflect the lack of access of pregnant women to primary education programmes on toxoplasmosis, dietary and hygiene measures, and recommendations to avoid seroconversion during pregnancy.

The seroprevalence of *T. gondii* infection in the population of pregnant women recorded in urban Cotonou has gradually decreased from 53.6% in 1993 [28], 48.9% in 2013 [22] and then 44.4% (95% CI 40.3–48.6) for the current study. This slight decrease in prevalence can be explained by the urban environment of this population, which in recent years has experienced an improvement in hygiene and public sanitation conditions that may contribute to the decrease in oocyst contamination. Despite this evolution, the prevalence in urban areas is far higher than in rural areas in Benin, with 36.1% in south-west Benin at Kpomassè [31] and 30.0% in north-west at Tanguiéta [9]. A meta-analytical study of research conducted in Benin over nearly three decades on toxoplasmosis supports this finding with a prevalence of 52.0% in urban areas versus 33.0% in rural areas [33]. Women residing in urban areas are therefore at higher risk of infection than those living in rural areas.

The study of risk factors indicates a statistically significant association, in multivariate analysis, between *Toxoplasma* seropositivity and epidemiology factors of *Toxoplasma* infection such as age time of pregnancy (with the age group 30–47 years), contact with cats and multigravidae women. In previous studies conducted in Benin, three risk factors were associated with seropositivity to *T. gondii* namely age, urban residence and consumption of raw vegetables [22, 31, 33]. The risk of occurrence of toxoplasmosis in pregnant women in Benin is multifactorial and may vary among the populations analyzed. Much more cautiously, the univariate analysis shows that some factors studied in the present survey reflect an association with the occurrence of *T. gondii* seropositivity at the threshold of significance, HIV seropositivity (*p* = 0.05) and the occupation of the pregnant woman as an office worker (*p* = 0.06). In similar studies conducted in Burkina Faso, HIV seropositivity strongly influences *Toxoplasma* seroprevalence in pregnant women [2], while professional occupation as an office worker is a new risk element recorded in our population and may be explained by the fact that some office workers sometimes eat poorly washed raw vegetables or food soiled by oocysts for lunch.

This seroprevalence rate implies that there are 54.6% (95% CI 50.5–58.8) non-immune women in this population at risk for primary *Toxoplasma* infection during pregnancy. These women should be counselled on toxoplasmosis prevention and undergo serological surveillance to detect possible primary infection in view of the very favourable environment for oocyst preservation and parasite circulation. Among them, only 19.8% of women benefited from a second serological control even though it was systematically proposed and the cost of the tests was covered in this study. This low compliance has several possible explanations: i) the majority of women did not have sufficient understanding of the consequences of congenital infection, ii) irregular prenatal consultations in this population [7], iii) the absence of a national strategy for systematic prenatal monitoring of non-immune women, or iv) the absence of a national consensus that makes pregnant women and healthcare providers indecisive and difficult to reach [37]. The lack of a national strategy does not allow for large cohort studies to estimate the true burden and impact of CT on populations [37], like with other diseases such as malaria or tuberculosis. In some healthcare systems such as in France and Austria [5, 26], there is a rigorous national surveillance strategy for pregnant women to diagnose possible primary gestational infections. In these healthcare policies, populations are informed of the threat of transplacental *Toxoplasma* infection and institute preventive and diagnostic measures.

In this study, no seroconversion was observed in the 19.8% seronegative women who had serological monitoring [16]. The IgG avidity test is of great interest for estimating the date of infection in a single sample according to the age of pregnancy in order to adapt the management of pregnant women and avoid unnecessary treatments [13, 25, 32]. The possibility of dating by IgG avidity, however, has limitations. It can only exclude infections less than 4 months old in the case of high avidity, as low or intermediate avidities can persist beyond 4 months. Assessing the date of infection according to gestational age is therefore difficult in African countries such as Benin or other countries where the date of first prenatal consultation is often late and follow-up irregular [1, 7, 21]. Thus, in our study, most women (72.3%) were included at the 2nd or 3rd trimester of pregnancy. These situations may be related to several sociocultural factors such as maternal age ≤ 18 years, primary and secondary level of education, lack of abortion history, lack of monthly income, lack of knowledge of the date of last menstrual period, and lack of awareness of beginning prenatal consultations [21].

Analysis of avidity results by gestational age detected 6 possible cases of per gestational infection, i.e., 1.1% (95% CI 0.5–2.0%) or 1,100 pregnant women with *T. gondii* infection during pregnancy per 100,000 pregnant women. This figure shows that a large number of newborns could be at risk for congenital infection if mothers are left without diagnosis and management of toxoplasmosis. This potential primary infection recorded late during pregnancy is not significantly higher than that reported in Kpomassè, Benin (0.5%) [31]. Rostami *et al.,* identified a high rate (1.6%) of primary infection acquired during pregnancy for Africa as a whole [30]. In Benin, an incidence of 3.4% of maternal primary infection was detected in the study cohort conducted between 2008 and 2010 in southern Benin in a semi-rural area [8]. This disparity may be due to the higher seroprevalence (52.6% *vs.* 44.4% in the current study), or to the fact that in the current study, seronegative women were educated about hygienic and dietary measures against toxoplasmosis during their pregnancy. However, the rate of possible primary infection in our study should not be considered as the true incidence of per gestational infection. It may be underestimated due the small percentage of seronegative pregnant women who underwent a second serology and to the fact that a large proportion received their first test only late in pregnancy. Above all, a larger sample size and a systematic control of seronegative women would have been necessary to determine the true incidence of this infection in pregnant women. The diagnosis of primary infection during pregnancy followed by chemoprevention with spiramycin, combined with neonatal work up and postnatal follow-ups for the first nine months of life, confirmed the absence of CT in the newborns. Several studies in Benin have estimated the existence of CT to concern between 200 [8] and 340 [35] infected newborns per 100,000 births. Numerous studies have reported that prenatal screening accompanied by primary health education measures and case management are of great benefit in controlling CT and limiting the consequences [6, 38] compared to neonatal screening.

### Conclusion

The prospective and analytical study conducted in an urban setting in Benin revealed an estimated seroprevalence of toxoplasmosis of 44.4% (95% CI 40.3–48.6) with an association of potential multifactorial risks. The increase of prevalence with age among adults demonstrates that infection is not uncommon among adults, and that pregnant women might be exposed, which differs from other settings in Africa where most infections are acquired in childhood. Pregnant women, especially the younger ones who are less likely to be immunised, should be educated on how to avoid contamination with oocysts or cysts.

## Supplementary material

The Supplementary materials of this article are available at https://www.parasite-journal.org/10.1051/parasite/2023040/olm *Survey questionnaire*: Toxo One Health Benin Project: Epidemiological survey questionnaire on toxoplasmosis in pregnant women.
