# Correction Notice to: “Seroepidemiology of toxoplasmosis in pregnant women and detection of infection acquired during pregnancy in Cotonou, Benin”: Parasite **30**, 43 (2023), https://doi.org/10.1051/parasite/2023040

**DOI:** 10.1051/parasite/2023050

**Published:** 2023-11-15

**Authors:** Richard Amagbégnon, Celia Dechavanne, Magalie Dambrun, Urielle Yehouénou, Noé Akondé, Florence Migot-Nabias, Aretas Babatoundé Nounnagnon Tonouhéwa, Azra Hamidović, Nadine Fievet, Angéline Tonato-Bagnan, Aurore Ogouyemi-Hounto, Maroufou Jules Alao, Marie-Laure Dardé, Aurélien Mercier, Dorothée Kindé-Gazard

**Affiliations:** 1 Centre Hospitalier Universitaire de la Mère et de l’Enfant-Lagune (CHU-MEL) 01 BP 107 Cotonou Bénin; 2 Institut de Recherche Clinique du Bénin (IRCB) Abomey-Calavi Benin; 3 Université Paris Cité, MERIT, IRD Paris France; 4 Unité de Recherche sur les Maladies Transmissibles (URMAT), Université d’Abomey-Calavi 01 BP 2009 Cotonou Bénin; 5 Inserm U1094, IRD U270, Univ. Limoges, CHU Limoges, EpiMaCT - Epidémiologie des maladies chroniques en zone tropicale, Institut d’Epidémiologie et de Neurologie Tropicale, OmegaHealth Limoges France; 6 Université d’Abomey-Calavi (UAC), Faculté des Sciences de la Santé (FSS) Bénin; 7 Service de Microbiologie du Centre National Hospitalier Universitaire – Hubert Koutoukou MAGA (CNHU-HKM) de Cotonou Bénin; 8 Centre National de Référence (CNR) sur la toxoplasmose/Toxoplasma Biological Resource Center (BRC), CHU de Limoges 87042 Limoges France

The article was published October 19, 2023 with an error in the order of authors’ names. Richard Amagbégnon is the first author, instead of Aurélien Mercier.

The Publisher will ensure that all modifications will be correctly transmitted to the indexation bases.

The Publisher apologizes for this mistake.

