# Supplementary material for: Seroepidemiology of toxoplasmosis in pregnant women and detection of infection acquired during pregnancy in Cotonou, Benin
Source: Parasite. 2023 Oct 19;30:43. doi: 10.1051/parasite/2023040 (PMC10586240; doi:10.1051/parasite/2023040)
Supplement: Supplementary file 1 — Survey questionnaire: Toxo One Health Benin Project: Epidemiological survey questionnaire on toxoplasmosis in pregnant women. [file parasite-30-43-s1.pdf]

Toxo One Health Benin Project: EPIDEMIOLOGICAL SURVEY QUESTIONNAIRE ON TOXOPLASMOSIS IN PREGNANT WOMEN

Survey n° : …………../ 1st letter last name–1st letter first name /EE Toxo One Health Benin

CPN file n° :…………………………………………..

Date : ……/……/…………. Contact or other person to contact ……………………………….

I- Socio-demographic data

1- Full name :…..……………………………………………………………………………..

Age :…….years 3- gravidity index :…… 4- Gestational age in weeks of amenorrhea …. …WA

5- Family status : single ☐ married ☐ divorce ☐ legal separation ☐

6- Department of origin/ country…………………………………………...……………………………....

7- Department of residence :…………………………………………...…………………………………

8-Commune :………………………………………………………………………………………...

9-District :……………………………………………………………………………….......

10-City :…………………………………………………………………………………...........

II- Socio-economic data

1- Education

High school or more ☐ secondary ☐ Primary ☐ Illitrate ☐

2- Spouse's level of education

High school or more ☐ secondary ☐ Primary ☐ Illitrate ☐

3- Professional situation

Executive> BAC ☐ office worker ☐ trader ☐ craftswoman ☐ housewife ☐

4- Professional situation of the spouse

Executive > BAC ☐ office worker ☐ trader ☐ craftsman ☐ no profession ☐

5- Life as a couple: Yes ☐ No ☐

I- Awareness of toxoplasmosis

1- Ever heard of toxoplasmosis yes ☐ no ☐

If yes :

Source of information: health personnel yes ☐ no ☐ literature yes ☐ no ☐

press yes ☐ no ☐ other yes ☐ no ☐

Toxo One Health Bénin

2- Mode of contamination yes ☐ no ☐

If yes :

Contact with cat yes ☐ no ☐ food/ raw vegetables yes ☐ no ☐

Contact with ground: yes ☐ no ☐ consumption of undercooked contaminated meat: yes ☐ no ☐

3- Clinical signs in the fetus yes ☐ no ☐

4- Complications of congenital toxoplasmosis

spontaneous abortion yes ☐ no ☐ severe neurological complications: yes ☐ no ☐

ocular lesions : yes ☐ no ☐

II- Eating behaviour

1- Eating habits

consumption of :

undercooked neat: yes ☐ no ☐ badly washed raw vegetables yes ☐ no ☐

undercooked vegetables yes ☐ no ☐ no information : ☐

2- Healthy living

Consumption of poorly treated water yes ☐ no ☐

Hand washing before each meal yes ☐ no ☐ no information: ☐

III- Contact with cat yes ☐ no ☐

IV- HIV status

HIV serology : Negatif ☐ Positif ☐ Not done ☐

V- Influence of IPT with Sulfadoxine-pyriméthamine (SP) on toxoplasmosis

1st dose : ☐ date ……./……./20….

2nd dose : ☐ date……../……./20…

3rd dose : ☐ date……./……/20…

No information ☐

Other antimalarial drug used:……………………………..date………./……/20…

Investigator/ Name………………………………………………………….. Signature…………………

Toxo One Health Bénin
